# Analysis of a Study of Lead Wheel Weight Deposition and Abrasion in New Jersey

**DOI:** 10.1007/s11270-015-2646-5

**Published:** 2015-10-22

**Authors:** Robert A. Root

**Affiliations:** Fair Oaks, CA USA

**Keywords:** Lead wheel weights, Motor vehicle wheel balancing weights, Street sweeping, Lead, Antimony, Antimonious lead, Lead pollution, Lead poisoning, Street lead, Lead pollution

## Abstract

This paper analyzes the implications for children’s health of shortcomings in the methods and results of a study of lead in the environment, “Quantity of Lead Released to the Environment in New Jersey in the Form of Motor Vehicle Wheel Weights,” by the New Jersey Department of Environmental Protection (Aucott and Caldarelli, *Water, Air, & Soil Pollution, 223*, 1743–1752, 2012). The study significantly understates the amount of lead deposited in New Jersey streets as 12 metric tons per year and incorrectly concludes that only 40 kg per year of the lead from wheel weights is abraded into small particles. The 2012 New Jersey Department of Environmental Protection (NJDEP) study misleads regulators and the public into believing that little toxic particulate lead from abraded wheel weights occurs on the streets of New Jersey and by implication that little occurs elsewhere in the United States, thus minimizing the potential health risk that lead wheel weights may have to our nation’s children and indeed all of us.

## Introduction

Lead exposure is a health issue that should concern everyone, because exposure to lead is on the rise (Fischetti 2013) and can have irreversible effects on young children and lifelong deleterious effects on us all. Lead has been linked to impaired cognition, attention deficit hyperactivity disorder (ADHD), psychiatric disorders, high blood pressure, heart arrhythmia, kidney damage, and dementia in seniors (Spivey 2007). But few are aware that some 1000 metric tons per year of lead weights (Bleiwas 2006; EPA 2015a; Parker 2013) used to balance vehicle wheels are lost on US streets, where a large number of these lead weights will be abraded into small particles, most of it near intersections (Root 2000). A likely pathway is for pedestrians to step in lead-contaminated road grit and unknowingly transport lead particles into their homes, where young children can get it onto their hands and into their mouths.

In 2006, the EPA provided a grant to the New Jersey Department of Environmental Protection (NJDEP) to investigate “the quantity of lead from automotive wheel weights entering the New Jersey environment…and its importance in the overall cycle of anthropogenic lead in the state.” Their study, “Quantity of Lead Released to the Environment in New Jersey in the Form of Motor Vehicle Wheel Weights,” by Michael Aucott and Adriana Caldarelli, was published in *Water, Air & Soil Pollution* (Aucott and Caldarelli 2012).

The authors of the NJDEP study “attempted to replicate the findings of wheel weight loss presented in the referenced paper,” a study of mine about lead wheel weight loss in Albuquerque, NM, published in *Environmental Health Perspectives* (Root 2000). In spite of their intent, the study they conducted does not replicate my study. Furthermore, their study understates the amount of lead lost in New Jersey and incorrectly concludes that virtually none of the lead from wheel weights is abraded into small particles. This minimization is important because it leads regulators and the public to believe that very little toxic lead from abraded wheel weights occurs on the streets of New Jersey and by implication that little occurs elsewhere in the United States. This paper identifies flaws in their methods and results that refute their conclusions. In what follows, I analyze the study designs and results of the NJDEP road surveys and abrasion study and identify how, in spite of their stated intent, their study neither replicates my study in 2000 nor provides realistic estimates of the amount of lead wheel weights deposited on New Jersey roads or the amount of these weights that enters the environment as particulate lead.

### Comparison of NJDEP and Albuquerque Road Survey Designs

The NJDEP lead wheel weight study consisted of 23 “steady-state” surveys on three road types in Mercer County—commercial, mixed use, and connector—with 12 of these surveys along a 1-km length of the commercial road. Surveys were recorded separately for two subsections of the commercial road, one 0.4 km, and the other 0.6 km. Of the 257 lead wheel weights found on all three road types, 88 % occurred along this single 1-km segment of commercial road. It was assumed that “steady-state” road conditions existed at the time of the surveys, which occurred at intervals of 1, 3, 4, 5, 6, and 7 weeks. Results from the surveys were used to estimate the amount of lead deposited, the rate of deposition, and the rate of wheel weight abrasion on New Jersey roadways.

My study in 2000 of lead wheel weights in Albuquerque, on the other hand, had a more complex design not replicated in New Jersey (Root 2000). The focus of my study was a six-lane principal arterial 2.4 km in length. Two types of surveys were conducted along the entire length of this arterial: one steady-state survey and 20 biweekly surveys (one every 2 weeks for 40 weeks). A series of 28 daily surveys was conducted along the west side of a 0.6-km segment of the same arterial. Steady-state surveys of seven similar arterials within the city of Albuquerque were also conducted to ensure a representative sample of steady-state deposition of wheel weights. The steady-state surveys of eight arterials, totaling 19.2 km, were used to calculate the amount of lead lost on major Albuquerque thoroughfares. The biweekly surveys were used to calculate deposition and abrasion rates, and the daily surveys were used to demonstrate that the rate of wheel weight deposition is constant. A two-week-long degradation study was also conducted on a segment of the same arterial, about 0.7 km north of where the surveys occurred, to demonstrate that lead wheel weights are rapidly abraded in the street.

The 1-km-long commercial road segment where the NJDEP found most of their wheel weights passes through a business district of Trenton. This road has an estimated traffic volume of 15,000 to 20,000 vehicles per day and a speed limit of 35 MPH (NJDOT, personal communication). To replicate my study in 2000, the NJDEP should have chosen a representative number of New Jersey roads with comparable traffic volume and speed limit. Most of the Albuquerque surveys were along a six-lane principal arterial with a speed limit of 40 MPH and an average daily traffic volume of 45,000 vehicles per day. Traffic volume and speed are important factors because wheel weights are most apt to be lost from vehicles when there is a rapid change in momentum such as a stop at an intersection. The higher the speed limit, the more rapid the deceleration required to stop. Thus, the difference between the number of weights lost on streets with a speed limit of 35 MPH and streets with 40 MPH can be significant. And the number of wheel weights lost is of course directly related to traffic volume. It is notable that the NJDEP report did not mention either traffic volume or speed limit.

The NJDEP conducted a painted weight study intended to measure the rate of wheel weight abrasion, an alternative approach to the Albuquerque biweekly surveys. The study dispersed wheel weights of known mass that could be recognized by paint color and other distinguishing marks. Painted wheel weights were dispersed on seven occasions at six locations along each direction of the four-lane commercial road segment where the highest rate of weights was found during the road surveys. In all, 102 painted weights were dropped, 12 weights on each of four occasions and 18 on three other occasions. The painted weights were left to be abraded by vehicular traffic for 7 to 53 days. The net weight loss was determined for the weights found that could be matched to the weights dispersed.

The Albuquerque study included a degradation study to determine the rate at which wheel weights are abraded in the street. During this study, 120 lead wheel weights representing nine weight sizes were scattered every day for 14 days at the center of each of three lanes (a total of 360 wheel weights weighing 6.97 kg) along one side of the six-lane arterial.

### Analysis of NJDEP “Steady-State” Road Surveys

The NJDEP describes the surveys they conducted as “steady-state” surveys. But their results indicate that many of the surveys were conducted when the road was not at a steady state. To reach a steady state, lead wheel weights accumulate and the amount of lead abraded increases until the weights deposited and the particulate lead abraded come into equilibrium. The initial survey of a road is thus referred to as a steady-state survey because the amount of lead lost from vehicles and amount of lead abraded have reached equilibrium. In order to be at a steady state, a road must be free of disrupting factors such as street cleaning or recent collecting of weights. When a road is at a steady state, the amount of lead found is at its peak.

Because, by definition, the maximum quantity of wheel weights is found when the road is at a steady state, one would expect the results of the NJDEP’s 12 commercial road surveys to be clustered around a substantial number of weights or quantities of lead if the road was at a steady state. Rather, the number of weights found for the 0.4-km subsection ranges from 1 to 26 wheel weights, with a mean of 10.7 and a standard deviation of 6.9. Twenty-six and 19 weights were found during two of the surveys, but only 1, 3, 6, 7, or 8 weights were found during some of the other surveys. It is unreasonable to believe that this commercial road was at steady state because of the substantial variability in the number of weights found. By definition, a steady state should not be so variable. This failure to assure that the roads were at a steady state when sampled means that the “mean steady-state amounts” were underreported and thus misrepresented the amount of wheel weight lead deposited.

The NJDEP intended to use the number of weights found during its “steady-state” surveys to calculate a deposition rate. The annual deposition rate they calculated “corresponds to approximately 14 steady-state amounts,” which means a steady state was attained every 26 days. However, the surveys conducted at 1 and 3 weeks since the last survey could not be steady-state surveys. In addition, if the commercial road actually attained a steady state in 26 days, by definition, the number of wheel weights or amount of lead found thereafter (assuming the road remained undisturbed) would essentially flatline at or near its maximum amount and therefore not be suitable for calculating a deposition rate because the amount of lead found at 7 weeks would be essentially the same as at 4, 5, and 6 weeks. Moreover, the rate of abrasion and the number of days it takes to reach a steady state are inversely related: a steady state attained in 26 days necessitates an average abrasion rate of 3.85 % per day.

Another reason to question the NJDEP deposition rates and amounts of lead found is that New Jersey roads with curbs, storm drains, and a posted speed limit of 35 MPH or less—such as the commercial road where most of their weights were found—are required by the New Jersey Department of Transportation to be cleaned at minimum of once per month, weather and street surface conditions permitting (NJDOT n.d.). Routine sweeping of their major survey street helps explain why their “steady-state” survey results are so erratic. Had the NJDEP chosen a road with a speed limit greater than 35 MPH, one like the principal arterial studied in Albuquerque, they would likely have avoided interference from street sweepers because these arterial roads are required to be cleaned only once every two years.

The NJDEP also identified an abrasion rate based on these road surveys and reported that an abrasion rate of 5 % per day “is in approximate agreement with the 2.72 % per day estimated by Root.” In fact, these two abrasion rates are substantially different: at 5 % per day a steady state is reached in 20 days while at 2.72 % per day it takes 36 days to reach a steady state.

### Analysis of NJDEP Painted Weight Study

The painted weight study, which attempted to measure the net weight loss of individually marked wheel weights exposed to the grinding of traffic in a four-lane road, identified an abrasion rate of 0.04 % per day, drastically different from the 5 % abrasion rate estimated during their road surveys. Some major problems in the painted weight study make the accuracy of the 0.04 % per day abrasion rate unlikely:

1. *Failure to account for missing weights in painted weight study conclusions*. According to the NJDEP, “most of the weights released were subsequently found…providing a way to measure the amount of weight that had been lost due to abrasion by traffic during the period in which the weights were in the road.” In fact, only 52 of the 102 painted wheel weights released were included in the abrasion analysis. The total net weight loss of these 52 weights was 10.3 g. According to the investigators, “the possibility that some weights disappeared entirely due to the abrasion and grinding of traffic appeared remote” even though their “steady-state” road surveys suggested that “approximately 5 % per day of the mass of the weights is lost from roads each day.” The failure to measure or even estimate the abrasion suffered by the 50 weights not included in the study analysis constitutes a fatal defect in the design and analysis of the study.
2. *Conclusion based on observations without data.* To account for missing weights, the NJDEP study concluded that “there are other ways besides grinding by traffic that weights are lost from road surfaces, and that these other ways represent a much larger component of the loss process….a major route of loss of weights is that they are eventually flung or knocked into culverts and other drainage structures…and that it is likely that weights are moved considerably by traffic and are likely to be eventually flung out of the path of vehicles.” These observations are interesting—but nothing in their study provides any evidence of “a larger component” than abrasion that explains how wheel weights are lost. While it is true that a few weights are flung beyond the curb and some eventually are washed into street drains, the vast majority of wheel weights are abraded in the roads where they are lost (Root 2000). Most wheel weights, which are lost from wheel rims along incoming lanes at traffic intersections, are flipped like tiddlywinks as vehicles run over them, and they rapidly accumulate where most are found: in the gutter. A small percentage is flipped onto the adjacent sidewalk; if a high percentage were flipped out of the street, they would accumulate and be found in larger numbers beyond gutters. But that is not what occurs. Street drains are usually mid-block or near the corner of the intersection, and a few weights are probably swept by stormwater into these drains. But since lead is about twice as dense as other street grit, it would likely take a torrent of stormwater to move many of them very far.
3. *Street sweeping as a reason for disappearance.* The study design failed to anticipate that the street where the study was conducted was cleaned monthly by street sweeping. Street sweeping is a means other than abrasion that results in the disappearance of lead wheel weights from roads. Chapter 7 of the New Jersey Department of Transportation (2015) Highway Agency Stormwater Guidance states that “all county streets with curbs, storm drains, and a posted speed limit of 35 MPH or less shall be swept a minimum of once per month, weather and street surface conditions permitting,” similar, non-county streets are to be swept once per quarter; and the remaining streets swept once every two years. If the roads surveyed by the NJDEP (which have speed limits of 35 MPH) attained a steady state in 26 days as they estimated, street sweeping at the recommended rate of once per month would reduce the amount of lead abraded on streets by approximately 40 %. In streets swept quarterly, abrasion would be reduced approximately 15 % per quarter. These estimates assume that the street sweeping is 100 % effective at removing wheel weights. Street sweeping once a year of principal arterials would reduce the abrasion of lead wheel weights approximately 5 % per year.
4. *Discrepancy between abrasion rates of painted weight study and “steady-state” surveys.* As inconsistent as the NJDEP “steady-state” surveys of the commercial road are, the result of one of the surveys conducted along the 0.4-km subsection of the commercial road provides evidence that the abrasion rate of 0.04 % per day, derived from the painted weight study, is wrong. According to the NJDEP project database, during the “steady-state” survey on June 8, 2008, which occurred 20 days after the previous survey, eight of the 19 weights found were described as “abraded,” five had “no clip” and two were “broken,” which suggests these weights had also been abraded. One of the wheel weights was 12.2 cm long and described as “full length, very semi-circular shape, no clip, aged, dull.” This description is of a wheel weight that would have weighed 85 g when it was lost. Its field weight on June 8 was 18.39 g, roughly 80 % less than its new weight. (Wheel weights have historically come in standard 0.25 oz (7.1 g) increments, and using the length and width of abraded weights, one can determine the minimum a given wheel weight would have weighed when new. Very few wheel weights are damaged by normal use, which means that on the day they fall off wheel rims into the street each weighs essentially the same as the day it was affixed to the rim.) Another weight, 8.3 cm long, was described in the project database as “regular size clip, looks aged, dull.” The length of this weight indicates it is likely a weight that when new would have weighed 56.7 g. Its field weight on June 8 was 27.80 g, a 51 % weight loss. A set of three weights 1.8 to 3.1 cm long with intact clips was described as “roughed up or abraded.” Their lengths are of weights that when new would have weighed 14.2 g. These three weights had a combined field weight of 13.62 g, an average weight loss of 68 %.

The net weight loss of the 19 wheel weights found during the June 8 survey was actually 34.4 %, an abrasion rate of approximately 5.2 % per day. This rate is consistent with the abrasion rate of 5 % the NJDEP reported based on their road surveys and refutes the abrasion rate of 0.04 % per day they reported for their painted weight study. It also challenges their statement that “the possibility that some weights disappeared entirely due to the abrasion and grinding of traffic appeared remote.” On the basis of such flaws, the results of the painted weight study are so strongly biased that the conclusion that lead wheel weights are abraded at a rate of 0.04 % per day is essentially worthless.

### Extrapolation of NJDEP Findings to New Jersey and the United States

The NJDEP extrapolated its findings to New Jersey and the United States, concluding that 12 metric tons per year of lead from wheel weights are deposited on New Jersey roads. This value was based on the results of their surveys of three road types in one county that do not include any six-lane or eight-lane thoroughfares and is therefore not representative of New Jersey roads. They further suggested that the national rate of wheel weight deposition is approximately 480 metric tons per year, “approximately one-third of that estimated by the Root study.” It is preposterous that they extrapolated the results of their study, based principally on survey results of 1 km of road, to the entire country.

To provide some perspective, in 2006, the U.S. Geological Survey (USGS) conducted a stocks and flows analysis of lead-based wheel weights in the United States and estimated that in 2003 approximately 2000 metric tons of wheel weight lead were lost on the nation’s roadways (Bleiwas 2006). According to the EPA (2015a), “in 2003, 65,000 tons of lead wheel weights were in use in the United States and approximately 2000 tons [per year] of these weights were lost from vehicles into the environment. Voluntary actions on the part of the US auto manufacturers and a European Union ban [in 2005] on their use reduced the number of lead wheel weights, but they continue to be the predominant product in the tire replacement market.”

New Jersey is not among the seven states—California, Vermont, New York, Washington, Illinois, Maine, and Minnesota—that have banned the use of lead wheel weights. These seven states have a combined population of approximately 83.5 million, approximately 27 % of the US population of 308.7 million in 2010 (U.S. Census Bureau 2011). The population of New Jersey, the most urban state in the nation, was 8.8 million in 2010 or approximately 3.9 % of the 225.3 million who reside in the 43 states that currently do not regulate lead wheel weights, which means that about 3.9 % of the lead wheel weights that continue to be lost in the remaining 43 states are expected to be lost in New Jersey. According to Perfect Equipment, Inc., the largest American manufacturer of wheel weights, 50 % are made of lead and 50 % are lead-free (Parker 2013).

Based on the lead deposition rate determined for the United States by the USGS and embraced by the EPA, it is estimated that some 39 metric tons per year of lead from wheel weights are deposited on New Jersey roads, substantially more than the 12 metric tons reported by the NJDEP. The calculation of the annual amount of lead from wheel weights deposited on New Jersey roads is therefore as follows:

- 2000 metric tons/year of wheel weight lead deposited (Bleiwas 2006; EPA 2015a); half of weights are of lead (Parker 2013) = 1000 metric tons per year,
- New Jersey population is 3.9 % of the population in the 43 states that allow use of lead wheel weights,
- 3.9 % of 1000 metric tons = 39 metric tons/year of wheel weight lead deposited on New Jersey roads.

There is ample evidence to conclude that most wheel weights fall from vehicles when the vehicles decelerate at intersections since most weights are found in the gutters of the incoming lanes of heavily traveled urban streets (Root 2000). As discussed above, street sweeping can reduce the amount of particulate lead resulting from the abrasion of these lost wheel weights, but even weekly street sweeping cannot totally eliminate this source of particulate lead because wheel weights are continuously lost and rapidly abraded.

The NJDEP study concluded that “approximately 12 metric tons per year of lead in the form of wheel weights are deposited on New Jersey roadways and that approximately 40 kg per year of this lead enters the environment in the form of small particles formed from the abrasion and grinding action of traffic on weights deposited on roadways.” In other words, they estimate that just 0.33 % of the lead abraded from their low estimate of the wheel weights lost on New Jersey roadways enters the environment as small particles. This estimate is based principally on their painted weight study whose conclusions are essentially meaningless, as discussed above. Further, my analysis of their June 8 survey, whose duration was just 20 days, showed a mean mass loss of 34.4 %, an abrasion rate of 5.2 % per day. This rate is consistent with the deposition rate Aucott and Caldarelli report in their results section: “the deposition rate [of the steady-state surveys] corresponds to approximately 14 steady-state quantities deposited per year, suggesting that approximately 5 % of the mass of weights is lost from roads each day.”

A reliable estimate of the amount of lead lost and abraded in New Jersey cannot be based on the data reported in the NJDEP study because no roads with speed limits of greater than 35 MPH—the fast-moving, high traffic volume, stop-and-go thoroughfares with multiple stops—were not surveyed. It is these principal arterials where wheel weight is most apt to be lost. Moreover, in spite of the multiple surveys the NJDEP conducted of a short segment of one four-lane road, their study lacks data on the deposition and abrasion of a representative number of four-lane roads; it cannot be simply assumed that one short segment is representative of all four-lane New Jersey roads.

If 39 metric tons per year of lead wheel weights are lost in New Jersey (as calculated above), all New Jersey roads were swept once a month (a conservative condition), and the abrasion rate was 4 % per day (steady state attained in 25 days), then approximately 23 metric tons per year would enter the New Jersey environment as small particles rather than the NJDEP estimate of 40 kg. If the abrasion rate was 5 % per day (steady state is attained in 20 days) and all else remained the same, the amount of abraded lead would increase to 26 metric tons per year. Moreover, these estimates of abraded lead would increase when roads with speed limits of greater than 35 MPH, which are swept as infrequently as once every two years, are factored separately.

### Implications of Findings for Lead Exposure to Children

Ingesting lead particles is the typical route of lead exposure for children (ATSDR 2015), and no safe blood lead level in children has been identified (CDC 2013). Nevertheless, an estimated 535,000 US children age 1–5 years have blood lead levels of ≥5 μg/dL, the threshold for adverse health effects of lead exposure in young children (CDC 2013). Such a finding suggests that every effort should be made to protect children from all potential sources of exposure to lead.

A multiagency federal study on lead exposures in US children (Levin et al. 2008) found that ≥30 % of children do not have an immediate lead paint hazard. According to the EPA (2005), “There are sources [other than lead-based paint] which may contribute to elevated blood lead levels, perhaps significantly. These sources include certain products that contain lead (such as wheel weights).” Wheel weights pose a health risk because most are lost at major intersections where pedestrians are apt to step on abraded lead grit and transport it home on their shoes, where young children can get it onto their hands and into their mouths. “Lead is particularly dangerous to children because their growing bodies absorb more lead than adults do and their brains and nervous systems are more sensitive to the damaging effects of lead” (EPA 2015b). Had the EPA fulfilled the commitment it made six years ago to ban lead wheel weights (New York Times 2009), this source of lead pollution might no longer exist in the United States.

## Conclusion

This analysis demonstrates that the NJDEP estimate of the quantity of lead lost on New Jersey roads is low and that approximately 23 to 26 metric tons per year of lead rather than the NJDEP estimate of 40 kg enter the New Jersey environment as small particles from the abrasion of lead wheel weights. Several of the NJDEP “steady-state” surveys were not at a steady state; deposition and abrasion rates were principally based on a single 1-km-long, four-lane road. Moreover, the estimate of abrasion based on a painted weight study whose results are essentially worthless led to their impossibly low estimate of wheel weight abrasion. The failure to measure or even estimate the abrasion suffered by approximately half of the weights dispersed but not found or included in the painted weight study analysis constitutes a fatal defect in the design of the study. Sadly, the EPA’s *Integrated Science Assessment for Lead* (EPA 2013) uses the estimate of 0.04 % per day derived from the NJDEP painted weight study rather than the 5 % per day estimate derived from the NJDEP “steady-state” surveys.

Importantly, the NJDEP study fails to address the real issue: whether lead abraded from wheel weights contributes to the lead burden of the people of New Jersey, especially young children, and if so, to what extent? To determine if abraded particles of lead wheel weights, which are made of antimonious lead, contribute to the lead poisoning of children, a study should have been conducted using the diagnostic clue of the presence of antimony.

The New Jersey legislature has proposed a ban on lead wheel-balancing weights for all vehicles within the state, including passenger and commercial vehicles (Tire Busines.com 2015). The continued use of lead wheel weights is, of course, unnecessary; wheel weights made of steel and zinc are commercially available in the United States. Because of the lack of federal regulation, it is imperative that states act to protect children from the serious long-term effects of lead poisoning. We are all responsible for the welfare of the nation’s children and must therefore demand that regulations prohibiting the manufacture, processing, distribution in commerce, use, and improper disposal of lead wheel-balancing weights be enacted and enforced.
